# The relationship between the systemic inflammatory response, tumour proliferative activity, T-lymphocytic infiltration and COX-2 expression and survival in patients with transitional cell carcinoma of the urinary bladder

**DOI:** 10.1038/sj.bjc.6603415

**Published:** 2006-10-03

**Authors:** M Hilmy, R Campbell, J M S Bartlett, A-M McNicol, M A Underwood, D C McMillan

**Affiliations:** 1 Department of Surgery, Royal Infirmary University, Glasgow G31 2ER, UK; 2 Department of Pathology, Royal Infirmary University, Glasgow G31 2ER, UK

**Keywords:** bladder cancer, Ki-67, C-reactive protein, T-lymphocytes, COX-2, survival

## Abstract

The relationship between the systemic inflammatory response, tumour proliferative activity, T-lymphocytic infiltration, and COX-2 expression and survival was examined in patients with transitional cell carcinoma of the urinary bladder (*n*=103). Sixty-one patients had superficial disease and 42 patients had invasive disease. Cancer-specific survival was shorter in those patients with invasive compared with superficial bladder cancer (*P*<0.001). On univariate analysis, stratified by stage, increased Ki-67 labelling index (*P*<0.05), increased COX-2 expression (*P*<0.05), C-reactive protein (*P*<0.05) and adjuvant therapy (*P*<0.01) were associated with poorer cancer-specific survival. On multivariate analysis of these significant factors, stratified by stage, only C-reactive protein (HR 2.89, 95% CI 1.42–5.91, *P*=0.004) and adjuvant therapy (HR 0.29, 95% CI 0.14–0.62, *P*=0.001) were independently associated with poorer cancer-specific survival. These results would suggest that tumour-based factors such as grade, COX-2 expression or T-lymphocytic infiltration are subordinate to systemic factors such as C-reactive protein in determining survival in patients with transitional cell carcinoma of the urinary bladder.

Bladder cancer is the fourth most common malignancy in the Western World. In the UK, there are 12 500 new cases each year and 5000 deaths annually (CancerStats, 2002). The mortality from transitional cell carcinoma of the urinary bladder increases significantly with the progression of superficial to invasive disease. One of the common prognostic marker in clinical use is tumour grade, which is subject to considerable intra- and interobserver variation. Therefore, monitoring the possible progression of superficial transitional cell carcinomas constitutes a significant proportion of the general urological consultants’ workload.

It is now recognised that disease progression is dependent on a complex interaction of the tumour and the host inflammatory response (Balkwill and Mantovani, 2001; Coussens and Werb 2002; Vakkila and Lotze 2004). Recently, the systemic inflammatory response, as evidenced by elevated circulating concentrations of C-reactive protein, has been shown to be independently associated with poorer survival in patients with advanced cancer (O’Gorman *et al*, 2000; Forrest *et al*, 2003; Maltoni *et al*, 2005). There is also evidence that C-reactive protein has independent prognostic value in primary operable cancer (Ikeda *et al*, 2003; McMillan *et al*, 2003; Jamieson *et al*, 2005; Crumley *et al*, 2006; Lamb *et al*, 2006). Therefore, it would appear that the systemic inflammatory response is of considerable importance in the relationship between the tumour, the host and outcome in patients with cancer. Recently, we have reported that an elevated C-reactive protein was associated with poor cancer-specific survival in patients with bladder cancer independent of tumour stage and grade (Hilmy *et al*, 2005).

The basis of the independent relationship between an elevated C-reactive protein concentration and poor survival in cancer is not clear. There are a number of possible explanations. Firstly, that an elevated C-reactive protein identifies tumours capable of producing significant amounts of proinflammatory cytokines, in particular interleukin-6 (Kinoshita *et al*, 1999; McKeown *et al*, 2004) and therefore with the potential for more rapid growth of tumour cells (Jee *et al*, 2001; Trikha *et al*, 2003). Alternatively, C-reactive protein could directly impair immune function (Maccio *et al*, 1998; Du Clos and Mold, 2004; Canna *et al*, 2005) allowing unrestrained tumour growth and dissemination.

Precise localisation of pro-inflammatory cytokines such as interleukin-6 to tumour cells or inflammatory cells within the tumour, particularly in paraffin-embedded tissues, remains problematical (Canna *et al*, 2005). However, tumour proliferative activity has been reliably assessed using the Ki-67 labelling index in a variety of solid tumours, including bladder cancer (Blanchet *et al*, 2001; Habuchi *et al*, 2005). Also, infiltration of tumours with T-lymphocytes has been reliably demonstrated in a variety of solid tumours, including bladder cancer (Stavropoulos *et al*, 1998; Bevers *et al*, 2004).

Central to the local inflammatory response is cyclooxygenase-2 and increased expression has been shown to be associated with poor survival in a number of common solid tumours (Dannenberg *et al*, 2001; Dannenberg and Subbaramaiah, 2003) including bladder cancer (Shirahama *et al*, 2001; Shariat *et al*, 2003).

The aim of the present study was therefore to examine the relationship between the systemic inflammatory response (C-reactive protein), tumour proliferative activity (Ki-67), T-lymphocyte (CD4+, CD8+) infiltration, and COX-2 expression and cancer-specific survival in patients with transitional cell carcinoma of the bladder.

## METHODS

### Patients

A cross-sectional retrospective study of patients with biopsy-proven transitional cell carcinoma and with a measurement of C-reactive protein before transurethral resection of bladder tumour in Glasgow Royal Infirmary between 1992 and 2001 was carried out. Tumours were grouped according to whether they were superficial (pTa, pT1 and CIS) or muscle invasive (pT2–pT4). However, patients with pT1G3 were considered as muscle invasive tumours as they are recognised to have a significantly higher progression rate (Manoharan and Soloway, 2005). At this time, no patient showed clinical evidence of infection, or other inflammatory conditions. Tumour stage was assessed using the 1997 AJCC/UICC TNM classification (Sobin and Wittekind, 1997), and tumour grade was performed according to the 1999 WHO grading system (Busch and Algaba, 2002).

Routine laboratory measurement of patient's serum for C-reactive protein concentration was performed. The limit of detection of the assay was a C-reactive protein concentration lower than 5 mg l^−1^. The coefficient of variation, over the range of measurement, was less than 5% as established by routine quality control procedures. C-reactive protein measurement of greater than 10 mg l^−1^ was considered to indicate the presence of systemic inflammatory response (O’Gorman *et al*, 2000).

The Research Ethics Committee of North Glasgow NHS Trust approved the study.

### Immunohistochemistry

Blocks from the primary tumour were fixed in 10% buffered formalin in saline and embedded in paraffin wax. One representative block of tumour was selected for each patient. Serial individual sections (4 *μ*m) were cut and mounted on slides coated with aminopropyltriethoxysilane for the immuno-histochemistry of Ki-67, CD4+ and CD8+ T-lymphocytes and COX-2 expression.

### Ki-67

Sections were immunostained using a streptavidin biotin technique (Dako, Cambridgeshire, UK) as previously described (McNicol *et al*, 1997). The primary antibody for Ki-67 was mouse monoclonal antibody (Dako, Cambridgeshire, UK).

### CD4+ and CD8+ T-lymphocytes

Sections were immunostained using the peroxidase-based Envision technique (Dako, Cambridgeshire, UK) as described previously (Bromwich *et al*, 2003). The primary antibody for CD4 was mouse monoclonal (Vector, Peterborough, UK) and that for CD8 was mouse monoclonal (Dako, Cambridgeshire, UK).

### COX-2

Sections were immunostained using the biotinylated/streptavidin peroxidase complex technique (Dako, Cambridgeshire, UK) as previously described (Edwards *et al*, 2004). The primary antibody was human monoclonal antibody (Cayman Chemical Co., Annbor, Michigan, USA).

### Morphometry

The percentages of Ki-67-reactive tumour cells were evaluated at × 400 magnification by scoring a minimum of 1000 tumour cells in randomly selected fields (Ki-67 labelling index). Cases were counted by two observers and the highest score was chosen as the corresponding index.

Quantitative analysis of the lymphoid infiltrate was performed using point counting (Anderson and Dunnill, 1965) with a random sampling technique. With this method, the volume occupied by any given component (volume density) is expressed as a percentage of the total volume of the tissue. A 100-point ocular grid was used at × 400 magnification and 30 fields were counted per case for CD4+ and CD8+ immunopositive cells. Only fields within the tumour (including cancer cell nests and surrounding tissue stroma) were counted. Any normal tissue on the slide was excluded from the analysis.

Semiquantitative analysis of the COX-2 expression was scored using a weighted histoscore method (Kirkegaard *et al*, 2006). Histoscores were calculated from the sum of (1 × % cells staining weakly positive)+(2 × % cell staining moderately positive)+(3 × % cells staining strongly positive) with a maximum of 300. The mean of the two observers’ scores were used for the analysis as previously described (Edwards *et al*, 2004; Witton *et al*, 2004).

The observers (MH and RC) were blinded to the clinical outcome of the patient.

### Statistical analysis

Data are presented as median and range. For the purpose of analysis, the tumour Ki-67 labelling index, CD4+ and CD8+ T-lymphocyte counts and COX-2 expression were grouped by tertiles. The relationships between these and other variables were analyzed using the Mantel–Haenszel (*χ*^2^) test for trend and Spearman's rank correlation as appropriate.

Survival analysis was performed using the Cox proportional hazard model. Multivariate survival analysis was performed using stepwise backward procedure to derive a final model of the variables that had a significant independent relationship with survival. To remove a variable from the model, the corresponding *P*-value had to be greater than 0.10. Deaths up to 31 August 2005 have been included in the analysis. Analysis was performed using SPSS software (SPSS Inc., Chicago, IL, USA).

## RESULTS

The characteristics of patients with bladder cancer (*n*=103) that were grouped according to stage are shown in the Table 1. The majority of patients were male, over the age of 65 years, had superficial disease and were with elevated C-reactive protein concentration preoperatively. Patients with invasive bladder cancer were older (*P*<0.01) and had higher grade tumours (*P*<0.001), increased Ki-67 labelling index (*P*<0.001), increased tumour expression of COX-2 (*P*<0.001) compared with superficial disease. Also, patients with invasive disease had lower tumour infiltration of CD4+ (*P*<0.05) and CD8+ T-lymphocytes (*P*<0.01) and had evidence of a systemic inflammatory response (*P*<0.05). Patients with invasive bladder cancer received more additional therapy (*P*<0.05). In total, 22 patients had additional therapy (seven cystectomy, nine radiotherapy and six bacillus of Calmette and Guerin (BCG)). Cancer-specific survival was shorter in those patients with invasive bladder cancer compared with superficial bladder cancer (*P*<0.001).

The median follow-up of the survivors was 60 months. During the course of the study, 66 patients died, 42 patients of their cancer and 24 of intercurrent disease. On univariate analysis, stratified by stage, increased Ki-67 labelling index (*P*<0.05), increased COX-2 expression (*P*<0.05), C-reactive protein (*P*<0.05) and no adjuvant therapy (*P*<0.01) were associated with poorer cancer-specific survival (Table 2).

On multivariate analysis of these significant factors, stratified by stage, only C-reactive protein (HR 2.89, 95% CI 1.42–5.91, *P*=0.004) and no adjuvant therapy (HR 0.29, 95% CI (0.14–0.62, *P*=0.001) was independently associated with poorer cancer-specific survival. When C-reactive protein was excluded from the multivariate analysis, stratified by stage, only Ki-67 labelling index (HR 1.56, 95% CI 0.99–2.45, *P*=0.045) and adjuvant therapy (HR 0.41, 95% CI 0.19–0.86, *P*=0.019) were independently associated with poorer cancer-specific survival.

The inter-relationships between the clinicopathological characteristics are shown in Table 3. In all patients, tumour grade was directly associated with Ki-67 labelling index (*P*<0.001), COX-2 expression (*P*<0.001), CD4+ (*P*<0.01) and CD8+ (*P*<0.001) T-lymphocytes, but not with C-reactive protein (*P*=0.152). The Ki-67 labelling index was directly associated with COX-2 expression (*P*<0.001), CD4+ (*P*<0.001) and CD8+ (*P*<0.01) T-lymphocytes and, and also with C-reactive protein (*P*=0.039). The tumour COX-2 expression was directly associated with the tumour CD4+ (*P*<0.01) and CD8+ (*P*<0.05) T-lymphocytic infiltrate and weakly with C-reactive protein (*P*=0.068). The tumour CD4+ T-lymphocytic infiltrate was directly associated with CD8+ (*P*<0.001) but not with C-reactive protein (*P*=0.556). The tumour CD8+ T-lymphocytic infiltrate was not associated with C-reactive protein (*P*=0.892).

## DISCUSSION

In the present study, tumour grade and Ki-67 labelling index were associated with increased tumour COX-2 expression and infiltration by CD4+ and CD8+ T-lymphocytes in patients with transitional cell carcinoma of the bladder. However, on univariate analysis, only increased Ki-67 labelling index and COX-2 expression were significantly associated with poorer cancer-specific survival. However, neither of these tumour-based factors was independently significant when a marker of the systemic inflammatory response (C-reactive protein) was included in the survival analysis. Therefore, the present study examines, for the first time, the relationship between the preoperative systemic inflammatory response and tumour-based factors and suggests that the systemic inflammatory response is more closely related to outcome in patients with transitional cell carcinoma of the bladder.

These results are consistent with the superior prognostic value of C-reactive protein compared with tumour T-lymphocytic infiltration in patients with primary operable colorectal cancer (Canna *et al*, 2005). One possible explanation is that C-reactive protein can be measured with greater accuracy and precision than tumour-based factors. Alternatively, it may be that C-reactive protein plays a more pivotal role in the tumour–host relationship. C-reactive protein is recognised to be an activator of innate immunity and a modulator of adaptive immunity (Du Clos and Mold, 2004) and its elevation is a precursor to progressive involuntary loss of weight and lean tissue, which are believed to be key factors in determining cancer survival (McMillan *et al*, 1998; Kotler, 2000).

The results of the present study do not exclude nonmalignant causes of an elevated C-reactive protein in patients with transitional cell carcinoma of the urinary bladder. However, it is of interest that C-reactive protein concentrations above the threshold used in the present study (>10 mg l^−1^) are rare (<5%) in the general elderly population in the West of Scotland (O’Reilly *et al*, 2006).

Therefore, it may be that an elevated C-reactive protein concentration would be a useful marker of the host inflammatory response, in addition to other risk factors, to stratify patients into specific follow-up regimes. If further prospective studies show this to be the case, it may therefore reduce the workload and anxiety in those patients with superficial bladder cancer in whom continuous follow-up is the norm despite less than 50% probability of tumour recurrence.

These results are consistent with previous studies that have examined the prognostic value of Ki-67 labelling index (Habuchi *et al*, 2005). Similarly, in the largest studies, tumour COX-2 expression has been shown to be significantly associated with survival on univariate but not multivariate analyses (Shirahama *et al*, 2001; Shariat *et al*, 2003). However, to our knowledge there have been no studies that have examined the relationship between tumour CD4+ and CD8+ T-lymphocytic infiltration and cancer-specific survival in patients with bladder cancer.

The results of the present study are consistent with the concept that there is an active immune response to tumour cell proliferation in these patients and that proliferative activity and COX-2 expression may play a role in disease progression. It is therefore of interest that intravesical treatment of superficial carcinoma with BCG vaccine appears to reduce both disease recurrence and progression (Lamm, 2000). It may be that those patients with a high Ki-67 labelling index and COX-2 expression and a marked lymphocytic infiltration will have a better response to BCG (Alexandroff *et al*, 1999; O’Donnell, 2005).

In summary, the results of the present study shows that, in addition to grade and Ki-67 labelling index, both local (tumour COX-2 expression) and systemic (C-reactive protein) markers of the inflammatory response were associated with poor cancer-specific survival. However, on multivariate analysis only C-reactive protein had independent prognostic value in patients with transitional cell carcinoma of the urinary bladder.

## Figures and Tables

**Table 1:** Relationship between tumour stage and clinicopathological characteristics in patients with bladder cancer

	**Superficial (*n*=61)**	**Invasive (*n*=42)**	***P*-value**
Age group (⩽65/>65 years)	30/31	10/32	0.010
Sex (male/female)	44/17	26/16	0.277
*Tumour grade*			
G1/G2/G3	26/32/3	0/6/36	<0.001
Ki-67 (tertiles 1, 2, 3)	20/30/11	5/14/23	<0.001
COX-2 (tertiles 1, 2, 3)	31/25/5	4/9/29	<0.001
			
*% Tumour volume T-lymphocytes*			
CD4+ (tertiles 1, 2, 3)	25/21/15	10/13/19	0.022
CD8+ (tertiles 1, 2, 3)	26/21/14	9/13/20	0.005
			
C-reactive protein (⩽10/>10 mg l^−1^)	32/29	13/29	0.031
Adjuvant therapy (no/yes)	53/8	28/14	0.014
Cancer-specific survival (months)	109 (94–125)^a^	41 (24–58)^a^	<0.001

a Mean (95% CI).

**Table 2:** Relationship between clinicopathological characteristics and cancer-specific survival, stratified by stage, in patients with bladder cancer; univariate survival analysis

	**Patients (*n*=103)**	**Hazard ratio (95%CI)**	***P*-value**
Age group (⩽65/>65 years)	40/63	1.52 (0.76–3.02)	0.234
Sex (male/female)	70/33	0.72 (0.37–1.39)	0.322
*Tumour grade*			
G1/G2/G3	26/38/39	1.18 (0.57–2.48)	0.653
Ki-67 (tertiles 1, 2, 3)	34 (2–78)^a^	1.73 (1.11–2.72)	0.017
COX-2 (tertiles 1, 2, 3)	200 (100–300)^a^	1.95 (1.11–3.42)	0.020
			
*% Tumour volume T-lymphocytes*			
CD4+ (tertiles 1, 2, 3)	2.70 (0–12.30)^a^	1.26 (0.84–1.88)	0.260
CD8+ (tertiles 1, 2, 3)	2.20 (0.16–10.20)^a^	1.27 (0.87–1.86)	0.221
			
C-reactive protein (⩽10/>10 mg l^−1^)	45/58	2.36 (1.17–4.74)	0.016
Adjuvant therapy (no/yes)	81/22	0.36 (0.17–1.75)	0.006

a Median (range).

**Table 3:** Inter-relationships between the clinicopathological characteristics in patients with bladder cancer

	**Ki-67 labelling index (tertiles 1, 2, 3)**	**COX-2 (tertiles 1, 2, 3)**	**CD4+ (tertiles 1, 2, 3)**	**CD8+ (tertiles 1, 2, 3)**	**C-reactive protein (⩽10/>10 mg l^−1^)**
	***P*-value**	***P*-value**	***P*-value**	***P*-value**	***P*-value**
Tumour grade (G1/G2/G3)	<0.001	<0.001	0.009	<0.001	0.152
Ki-67 labelling index (tertiles 1, 2, 3)		<0.001	<0.001	0.002	0.039
COX-2 (tertiles 1, 2, 3)			0.005	0.019	0.068
					
*T-lymphocytes (% tumour volume)*					
CD4+ (tertiles 1, 2, 3)				<0.001	0.556
CD8+ (tertiles 1, 2, 3)					0.892
